# Resection of infected bulla associated with isolated unilateral absence of pulmonary artery: a case report

**DOI:** 10.1186/s44215-023-00122-6

**Published:** 2023-11-20

**Authors:** Megumi Kusano, Takashi Sakai, Takahiro Yoshizawa, Satoshi Koezuka, Yoko Azuma, Kazuma Kishi, Akira Iyoda

**Affiliations:** 1 Division of Chest Surgery, Department of Surgery, Toho University School of Medicine, 6-11-1, Omorinishi, Ota, Tokyo, 143-8541 Japan; 2 Division of Respiration, Department of Internal Medicine, Toho University School of Medicine, Tokyo, Japan

**Keywords:** Isolated unilateral absence of pulmonary artery, Infected bulla, Unilateral pulmonary artery defect

## Abstract

**Background:**

A unilateral absence of the pulmonary artery (UAPA) is a rare congenital anomaly, which in most patients is associated with congenital cardiovascular abnormalities. Some patients with UAPAs do not have associated cardiovascular abnormalities, called isolated UAPA. Patients with isolated UAPA generally have a mild clinical course and may not be diagnosed until adulthood, but repeated complications such as infections may make surgery difficult.

**Case presentation:**

A 34-year-old female patient with isolated right UAPA was referred to our hospital with a complaint of fever. Chest radiography and computed tomography showed an infectious cavity in the right upper lung. Surgery was scheduled for the patient; however, her preoperative condition needed to be optimized. She was evaluated for pulmonary hypertension and the collateral arteries were mapped out, and a waiting period after treatment of infection was set to avoid perioperative intrathoracic infections. Resection of the infected bulla was performed without any complications. No recurrence of infection was observed two years post-operatively. The residual lung expanded enough to fill the dead intrathoracic space.

**Conclusions:**

This report suggests the importance of adequate preoperative evaluation and perioperative management for patients with isolated UAPA who require surgical resection.

## Background

A unilateral absence of the pulmonary artery (UAPA) is a rare congenital anomaly, which in most patients is associated with congenital cardiovascular abnormalities [1]. However, some patients with UAPA do not have associated cardiovascular abnormalities, called isolated UAPA. These patients generally have a mild clinical course and may not be diagnosed until adulthood [2, 3]. Isolated UAPA in adults is a rare condition commonly associated with bullous lung changes and bronchiolitis, which sometimes requires surgical management due to hemoptysis and recurrent pulmonary infections [4]. Collaterals are well-developed, and pulmonary hypertension is often present in 44% of patients with UAPA [5]. Moreover, 35% of adult patients with UAPA have a recurrence of respiratory infections. Thus, intrathoracic adhesions are to be expected. Therefore, surgery should be planned carefully to prevent perioperative complications. Herein, we report a case of UAPA with an infected bulla, which has never been reported. The patient underwent resection of the inflected bulla with no complications due to thorough preoperative evaluation along with the surgical technique and method used to obtain infection control.

## Case presentation

**Fig. 1:**
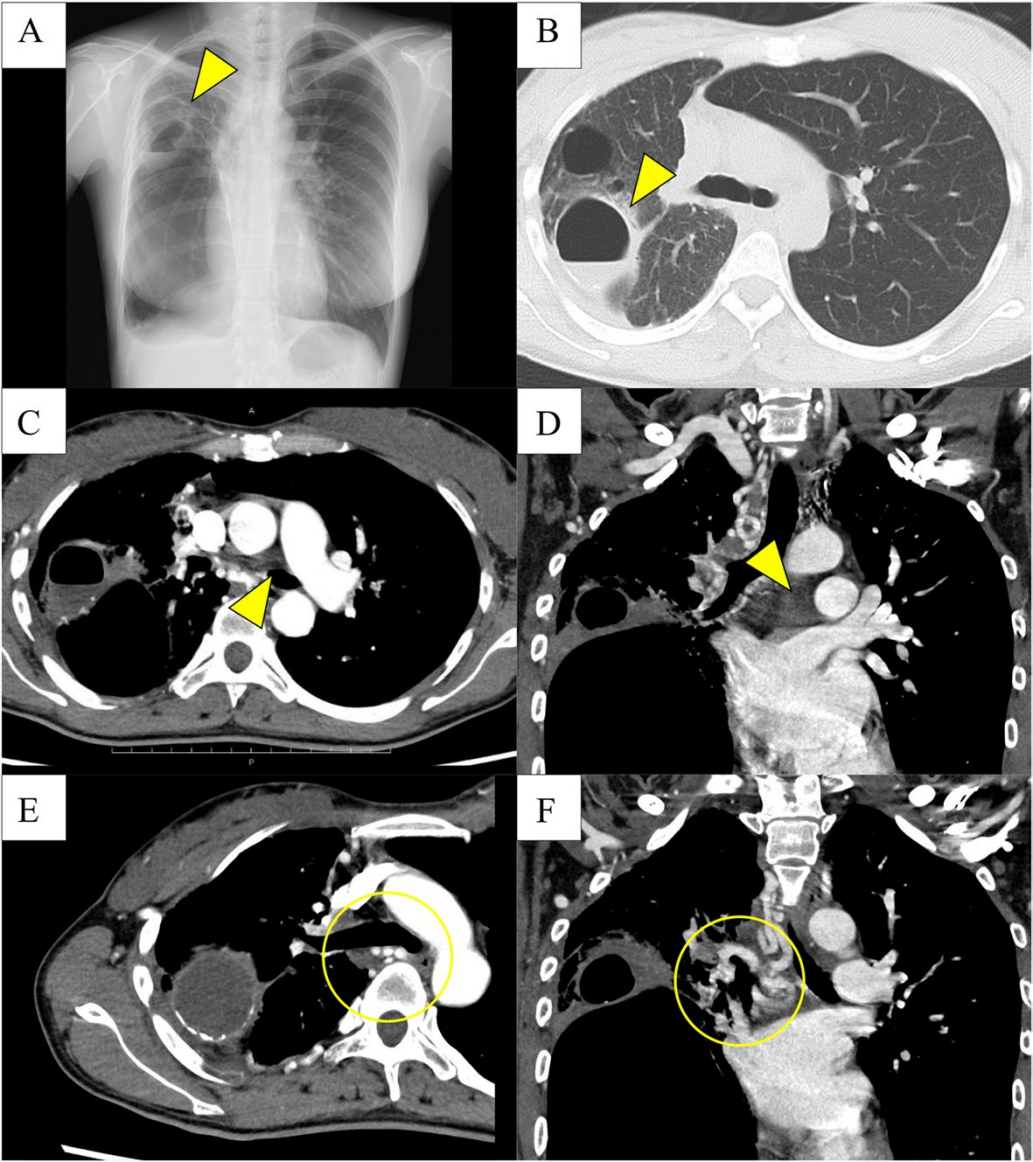
Perioperative imaging. **A** Chest radiography showing the fluid collection in one of the right lung bulla. **B** Computed tomography (CT) showing the cavity with the fluid collection and inflammatory changes around the cavity, suggesting an infection. **C**, **D** A contrast-enhanced CT scan confirming the absence of the right pulmonary artery. **E**, **F** Collateral arteries are shown to have developed from the bronchial arteries to the aortic arch

A 34-year-old female patient was referred to our hospital with a complaint of fever. She was diagnosed with an infectious lung cavity in the right upper lung on chest radiography and computed tomography (CT) (Fig. 1A and B). She was previously diagnosed with isolated UAPA, in which her entire upper right lobe was cystic, was regularly followed up at another hospital, and had been asymptomatic and healthy until this consultation (Fig. 1C and D).

**Fig. 2:**
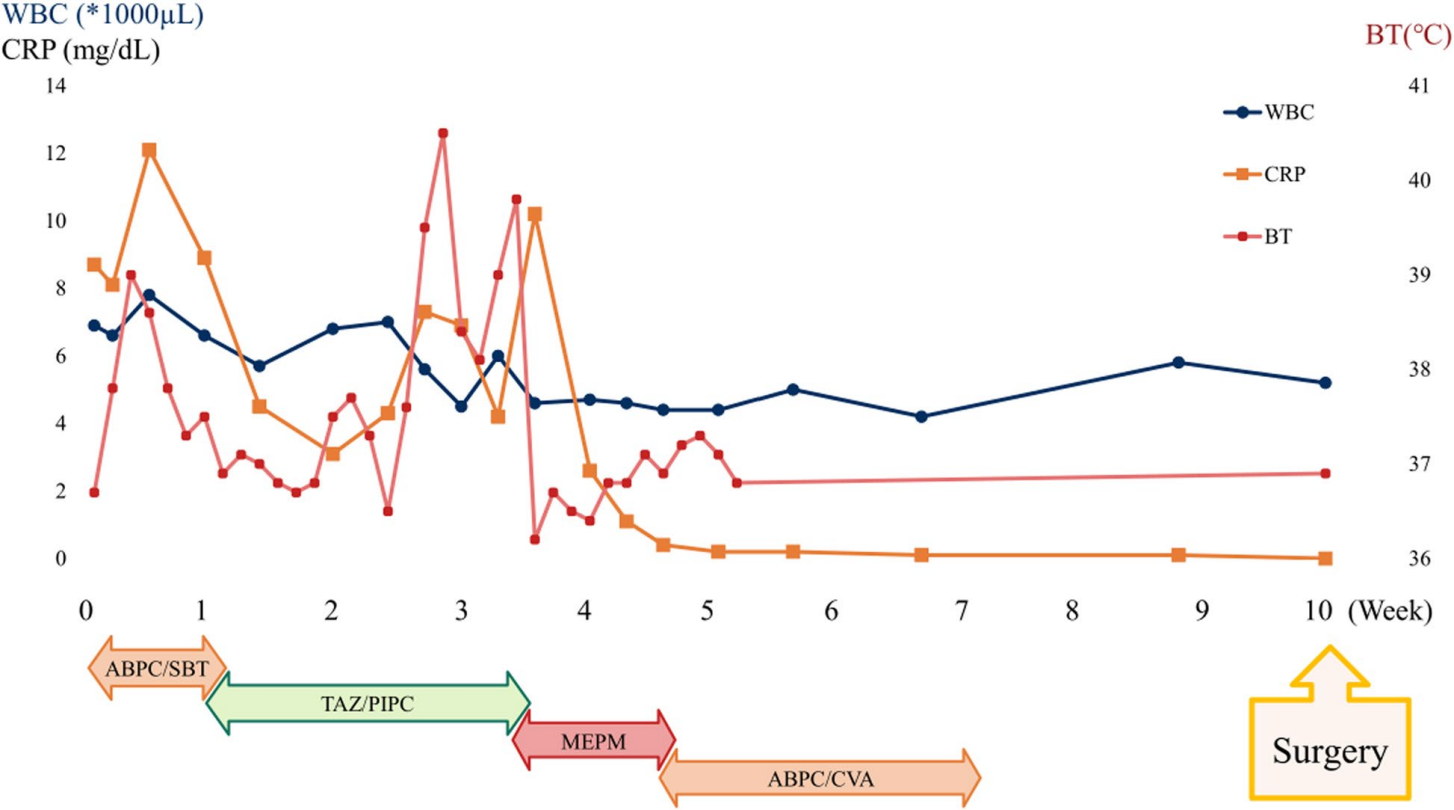
Chart of the body temperature, blood tests, and treatment course. After admission, three types of antibiotics were administered. The body temperature, white blood cell (WBC) count, and C-reactive protein levels did not improve until 3 weeks, and the length of hospitalization was 5 weeks. The surgery was performed 5 weeks after the discharge when tests for infection were normal to reduce adhesions and lung inflammation

The patient was treated with three types of antibiotics: ampicillin and sulbactam for 4 days, tazobactam and piperacillin for 10 days, and carbapenem for 10 days with no improvement in her symptoms or serum infective markers. On the 23rd day after the start of treatment, the patient coughed up a large amount of green sputum, after which a rapid decrease in the fluid level in the infected cavity was observed on chest radiography (Fig. 2). The patient’s condition gradually improved, and she was discharged 35 days after admission.

The patient was breastfeeding and had a high risk of recurrence and refractory UAPA infection; therefore, we planned for preventative surgical management. Several preparatory steps were taken to ensure the safety of the procedure. Firstly, a contrast-enhanced CT scan was obtained to evaluate the collateral arteries. There were multiple collateral arteries in the chest wall, diaphragm, and mediastinum. Bronchial arteries branching from the descending aorta were detected around the main right bronchus and pulmonary veins (Fig. 1E and F). Secondly, her cardiac function, including the presence of pulmonary hypertension, was assessed to be normal. Thirdly, given that active pulmonary infection is a risk factor for postoperative infectious complications such as empyema, we closely followed up with the patient for 5 weeks after discharge, during which the tests for infection assessment were normal (Fig. 2).

**Fig. 3:**
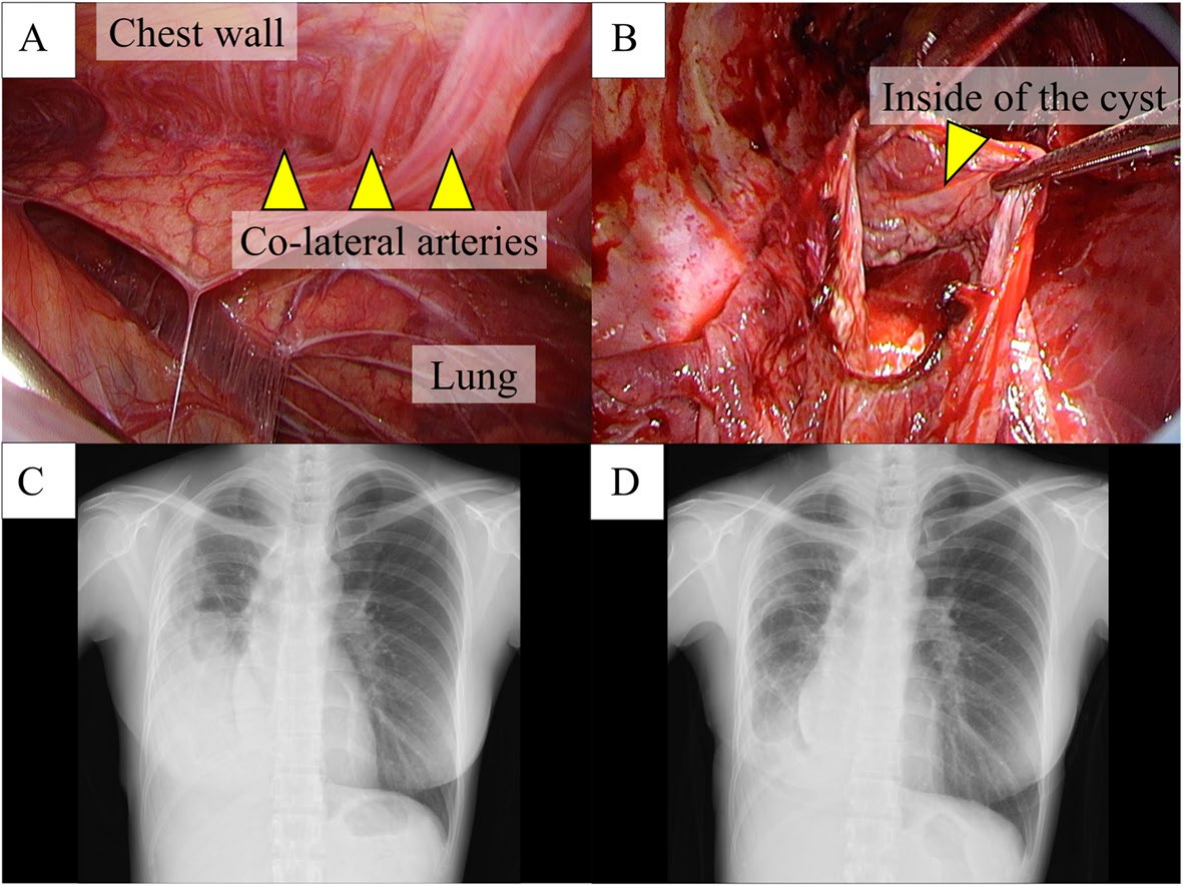
**A** Intraoperative findings of the bullae resection. Several collateral arteries from the chest wall were flowing into the lung, making adhesiolysis difficult. **B** Both the infectious bulla and the giant bulla attached to it were resected. We opened the giant bulla to confirm the boundary with normal lung tissue from inside the bulla and resected the bulla using a stapler via video-assisted thoracic surgery. The dead intrathoracic space in the right thoracic cavity remained after the surgery. The remaining lung gradually expanded without the appearance of new bullous change with the resolution of the dead space (**C**, **D** 3 and 6 months later, respectively)

We performed a two-port video-assisted thoracic surgery with 6- and 1-cm port sites. Although severe adhesions with numerous collateral arteries from the chest wall made the surgery difficult (Fig. 3A), the resection of the infected bulla was performed successfully with a blood loss of 261 ml and a surgical time of 4 h. The bleeding mainly occurred from the chest wall during the adhesiolysis and could be controlled by coagulation. Although we did not want to leave a large dead space (Fig. 1A), we had to resect both the infected and noninfected bullae because of the adhesions between the two cavities (Fig. 3B). The patient was discharged five days post-operatively without any complications and showed no recurrence of infection for two years. The intrathoracic dead space in the right thoracic cavity remained immediately after the surgery; however, the remaining part of the lung gradually expanded, and the dead space almost disappeared (Fig. 3C and D, 3 and 6 months postoperatively, respectively).

## Discussion

Most cases of UAPA are symptomatic from birth or early childhood, but approximately 30% remain asymptomatic until adulthood [6]. Symptoms include recurrent infections and bloody sputum. Some develop hemoptysis and infections that require surgical management [7, 8]. In addition, the affected lung is hypoplastic due to the absence of the pulmonary artery, resulting in bronchiectasis and cystic changes, leading to infection of these cysts or the development of pneumothorax, requiring surgery [4]. There are some reports of lung cancer associated with UAPA [9]. Thoracic surgeons are usually the ones tasked to operate on patients with UAPA.

There are many things to be aware of when operating on a patient with UAPA: Firstly, the development of collateral arteries in the affected lung. Various arteries, such as the bronchial, inferior diaphragmatic, internal thoracic, intercostal, and coronary arteries, have been reported as sources of inflow to the collateral arteries in UAPA [6]. Surgery must be performed with care because intraoperative injuries of the collateral arteries may be critical [1, 2]. Secondly, surgeons should be aware of the possibility of intrathoracic adhesions. Repeated infections often result in severe adhesions, and the process of removing adhesions while addressing the collateral arteries is extremely challenging. Thirdly, the risk of pulmonary hypertension, which is reported to occur in approximately 28% of adults with UAPA, should be considered [10]. Patients with pulmonary hypertension are at risk of severe perioperative complications; thus, preoperative evaluation of pulmonary hypertension is essential for patients with UAPA.

In this patient, collateral arteries were thoroughly investigated preoperatively. The collateral arteries were present in all directions from the mediastinum and chest wall and were expected to require careful surgical manipulation. Therefore, we avoided excessive debridement and performed partial resection of the infected bulla to prevent bleeding. Moreover, by minimizing resection to eliminate the dead space and controlling the infection sufficiently before surgery, empyema and intrapulmonary infection could be prevented. Although infected bulla in UAPA patients is extremely rare, we may have the opportunity to perform surgery for UAPA patients to prevent recurrent pulmonary infections. In such cases, the aforementioned surgical techniques may provide favorable outcomes.

## Data Availability

The case report and patient consent form is available.

## Abbreviations

CT: Computed tomography
UAPA: Unilateral absence of the pulmonary artery

## References

[1] Pfefferkorn JR, Löser H, Pech G, Toussaint R, Hilgenberg F. Absent pulmonary artery. A hint to its embryogenesis. Pediatr Cardiol. 1982;3(4):283–6. 6761655 10.1007/BF02427028

[2] Bockeria LA, Makhachev OA, Khiriev TK, Abramyan MA. Congenital isolated unilateral absence of pulmonary artery and variants of collateral blood supply of the ipsilateral lung. Interact Cardiovasc Thorac Surg. 2011;12(3):509–10. 21345836 10.1510/icvts.2010.250795A

[3] Griffin N, Mansfield L, Redmond KC, Dusmet M, Goldstraw P, Mittal TK, et al. Imaging features of isolated unilateral pulmonary artery agenesis presenting in adulthood: a review of four cases. Clin Radiol. 2007;62(3):238–44. 17293217 10.1016/j.crad.2006.10.006

[4] Kruzliak P, Syamasundar RP, Novak M, Pechanova O, Kovacova G. Unilateral absence of pulmonary artery: pathophysiology, symptoms, diagnosis and current treatment. Arch Cardiovasc Dis. 2013;106(8):448–54. 23938302 10.1016/j.acvd.2013.05.004

[5] Ten Harkel A, Jan D, Blom NA, Ottenkamp J. Isolated unilateral absence of a pulmonary artery: a case report and review of the literature. Chest. 2002;122(4):1471–7. 12377882 10.1378/chest.122.4.1471

[6] Wang P, Yuan L, Shi J, Xu Z. Isolated unilateral absence of pulmonary artery in adulthood: a clinical analysis of 65 cases from a case series and systematic review. J Thorac Dis. 2017;9(12):4988–96. 29312703 10.21037/jtd.2017.11.49 PMC5756977

[7] Ohtsuka T, Nomori H, Watanabe K, Kaji M, Ebihara A, Naruke T, et al. Isolated unilateral absence of a pulmonary artery treated by pneumonectomy in an adult: report of a case. Surg Today. 2006;36(6):525–7. 16715422 10.1007/s00595-006-3182-0

[8] de Mello WT Junior, Coutinho Nogueira JR, Santos M, Pelissari França WJ. Isolated absence of the right pulmonary artery as a cause of massive hemoptysis. Interact Cardiovasc Thorac Surg. 2008;7(6):1183–5. 18775917 10.1510/icvts.2008.180430

[9] Wang J, Lu X, Ding X, Cao D. Left lung cancer in a patient with congenital unilateral absence of the left pulmonary artery: a case report and literature review. World J Surg Oncol. 2020;18(1):32. 32028965 10.1186/s12957-020-1810-6 PMC7006177

[10] Shakibi JG, Rastan H, Nazarian I, Paydar M, Aryanpour I, Siassi B. Isolated unilateral absence of the pulmonary artery. Review of the world literature and guidelines for surgical repair. Jpn Heart J. 1978;19(3):439–51. 691278 10.1536/ihj.19.439

